# The Relationship between Chemokine Ligand 3 and Allergic Rhinitis

**DOI:** 10.7759/cureus.7783

**Published:** 2020-04-22

**Authors:** Ovidiu Berghi, Mihai Dumitru, Ramona Caragheorgheopol, Catalin Tucureanu, Anca Simioniuc-Petrescu, Roxana Sfrent-Cornateanu, Calin Giurcaneanu

**Affiliations:** 1 Dermatology, Carol Davila University of Medicine and Pharmacy, Bucharest, ROU; 2 Anatomy, Carol Davila University of Medicine and Pharmacy, Bucharest, ROU; 3 Immunology, Immunology Laboratory, "Cantacuzino" National Institute for Military Medical Research and Development, Bucharest, ROU; 4 Immunology, University of Bucharest, Bucharest, ROU; 5 Ambulatory, Sfânta Maria Clinics and Laboratories, Bucharest, ROU; 6 Immunology and Pathophysiology, Carol Davila University of Medicine and Pharmacy, Bucharest, ROU; 7 Oncologic Dermatology, Carol Davila University of Medicine and Pharmacy, Bucharest, ROU

**Keywords:** chemokine, allergic rhinitis

## Abstract

Background

Allergic rhinitis (AR) is a chronic and frequent condition characterized by an excessive response of the immune system to innocent substances encountered in the nasal mucosa. These reactions are mediated by many factors, including chemokines. Chemokine ligand 3 (CCL3, a macrophage inflammatory protein 1α) is a chemokine implicated in the activation of mast cells - white cells shown to be highly involved in orchestrating allergic reactions. The present study evaluated the role of CCL3 in AR.

Material and methods

Thirty-nine participants, including 24 patients with AR and 15 healthy controls, were evaluated for allergies to dust mites, cat and dog danders, cockroaches (Blatella germanica), molds, grasses, weeds, and tree pollen using skin prick tests. Participants were also evaluated for inflammatory conditions by measuring total blood count with differential; concentrations of rheumatoid factor, fibrinogen, and C-reactive protein; and erythrocyte sedimentation rate. CCL3 in blood samples was measured at the Immunology Laboratory, Cantacuzino National Institute for Military Medical Research and Development, Bucharest, Romania, using Human Multianalyte Profiling Base Kits (R&D Systems Inc., Minneapolis, MN).

Results

Mean serum CCL3 concentration was significantly higher in patients with AR than in controls (15.03 ± 7.11 pg/ml vs. 8.34 ± 4.46 pg/ml, p = 0.001 [t-test] and p = 0.026 [Mann-Whitney test]). CCL3 concentrations correlated with polysensitization, defined as two or more positive prick tests per patient (r = 0.325, p = 0.046) and seasonal AR (r = 0.482, p = 0.002).

Conclusions

Elevated levels of CCL3 were seen in our patients with AR. We have observed correlations with polysensitization and seasonal allergies. These results suggest that chemokines might play an important role in the pathogenesis of AR. In the future, chemokines might be used in endotype classification of patients with AR and as a possible target in the treatment of AR.

## Introduction

Allergic rhinitis (AR) is a symptomatic condition of the nose induced by an immunoglobulin E (IgE)-mediated inflammatory reactions after exposure of the nasal mucosa to an allergen. Chemokines play an important role in both the immediate and late phases of the allergic inflammatory process, inducing the activation and migration of immune system cells, including mast cells and eosinophils, into target organs, and activating macrophages to trigger B-cells to synthesize allergen specific-IgE [1]. Chemokine ligand 3 (CCL3, a macrophage inflammatory protein 1α) is produced by a variety of cells, including lymphocytes, resident and recruited monocytes and macrophages, fibroblasts, and epithelial cells. CCL3 is a potent chemokine that binds to and activates lymphocytes and monocytes through its receptors chemokine receptor (CCR)1 and CCR5. CCL3 synthesis is important in maintaining the recruitment of inflammatory cells during episodes of inflammation, as well as activating eosinophils and T-cells and regulating Ig production [2,3]. CCL3 constitutes a second signal for mast cell degranulation, acting as a direct costimulatory signal via CCR1. CCL3 is, therefore, essential for inducing acute-phase AR, making this chemokine critical for mast cell activation [4].

Several studies in humans have investigated the role of CCL3 in respiratory allergies. A pharmacological study investigating the role of the antihistamine fexofenadine found that nasal antigen challenge increased peak levels of CCL3 in nasal secretions, from 8.1 ± 1.8 ng to 11.2 ± 1.7 ng (P = 0.05) [5]. Levels of CCL3 increased observed six to eight hours after challenge with the nasal allergen Timothy grass pollen and decreased after treatment with fluticasone propionate, an inhibitor of Th2 cytokine synthesis [6]. The levels of CCL3 were significantly higher in patients with seasonal allergic rhinitis than in patients with perennial allergic rhinitis and healthy controls [3]. Moreover, serum concentrations of CCL3 in subjects allergic to ragweed were reduced outside the pollen season [7]. High levels of CCL3, along with high levels of the chemokines eotaxin, RANTES (Regulated upon Activation, Normal T-cell Expressed and Presumably Secreted), and monocyte chemoattractant protein-1 were observed during late responses to grass pollen [8]. Also, high levels of CCL3 messenger RNA were induced in mouse macrophage RAW 264.7 cells by birch and oak dusts, which have been associated with the development of allergic diseases [9]. The present study compared serum concentrations of CCL3 in patients with AR and healthy controls and correlated these concentrations with various aspects of respiratory allergies.

## Materials and methods

This prospective study was approved by the local medical committee of Santa Maria Clinics and Laboratories (formerly Anima Medical Center Bucharest) and the Ethics Committee for Research of Carol Davila University of Medicine and Pharmacy Bucharest. All participants provided written informed consent before enrollment.

This study enrolled 39 participants, 24 patients with AR and 15 healthy controls, all aged >18 years. AR was diagnosed using the 2019 updated Allergic Rhinitis and its Impact on Asthma criteria [10]. Twenty patients were diagnosed as having seasonal allergic rhinitis (SAR) and four perennial allergic rhinitis (PAR).Seasonal allergies usually occur during the spring and fall season in response to outdoor allergens like pollens.Perennial allergies can occur year round in response to indoor allergens, like dust mites, cockroaches and pet dander. Allergic sensitization was assessed according to the European Academy of Allergy and Clinical Immunology guide for the investigation of respiratory allergies [11]. All participants were subjected to skin prick tests (Lofarma, Milan, Italy) for 22 aeroallergens considered relevant to Europe, including Romania. These included dust mites (Dermatophagoides farinae and D. pteronyssinus), cockroaches (Blatella germanica), cat and dog danders, molds (Alternaria, Aspergillus, Penicillium and Cladosporium), and pollens of grasses, cereals, weeds (Ambrosia, Artemisia, Parietaria, Plantago lanceolata, and Helianthus annuus) and trees (Betulaceae, Oleaceae, Platanus occidentalis, Cupressaceae, Fagaceae, and Salicaceae). All tests were performed by an allergist, with a positive result defined as a wheal diameter ≥3 mm with surrounding erythema. All tests included histamine dihydrochloride (1 mg/ml) as a positive control and allergen-free saline solution as a negative control. The severity of AR was evaluated using the Total Nasal Symptom Score (TNSS), which is composed of at least three of the following four nasal symptoms: rhinorrhea, nasal congestion, nasal itching, and sneezing, with each having three levels of severity (mild, moderate and severe) [12]. Other inflammatory conditions were excluded by serologic analysis, including total blood count with differential; concentrations of rheumatoid factor, fibrinogen, and C-reactive protein; and erythrocyte sedimentation rate. Only participants with normal values were included in the study. All participants were examined by an otorhinolaryngologist using a flexible nasal fibroscope, with nasal mucosal hypertrophy (NMH) considered a sign of chronic inflammation.

CCL3 concentrations in blood samples were measured at the Immunology Laboratory of the Cantacuzino National Institute for Military Medical Research and Development Bucharest, using Human Multianalyte Profiling Base Kit A (R&D Systems Inc., Minneapolis, MN). The measurement of CCL3 levels in serum samples was realized in our study because in our facilities (clinics and laboratory) only for blood products were possible sampling, transport, storage and analysis of the tests. The detection limit was set to 3.2 pg/ml according to manufacturer’s instructions. Plates were read on a Luminex 200 platform (Luminex Corporation, Austin, TX), and data were processed with Luminex 200 IS 2.3 Star Station software (Applied Cytometry, Plano, TX) [13]. Concomitantly we have measured the serum levels of other chemokines (MCP-1/CCL2 (monocyte chemoattractant protein-1), IP-10/CXCL10 (interferon gamma-induced protein 10), ENA-78/CXCL5 (epithelial-derived neutrophil-activating peptide 78)) in order to obtain a wider image of the role of different subfamilies of chemokines in the pathogenesis of AR. We did not reach a statistical significance.

All statistical analyses were performed using IBM SPSS Statistics for Windows, Version 20.0. (IBM Corp., Armonk, NY) [11]. Because the groups were unbalanced, they were compared by Mann-Whitney U test groups with the Monte Carlo simulation technique and Student’s t-tests (for equal variances not assumed). The correlation of variables was assessed by Pearson’s or Spearman’s correlation test, as appropriate. The 95% reliability range of all data were reported, and p-values <0.05 defined as statistically significant.

## Results

The baseline demographic and clinical characteristics of the study subjects are detailed in Table 1.

**Table 1:** Baseline demographic and clinical characteristics of the study population AR: Allergic rhinitis; M/W: Men/women; IgE: Immunoglobulin E; VEMS: Maximum expiratory ventilation per second; N.V.: Normal values.

Characteristic	AR patients	Healthy controls
Number	24	15
Age (median), years	33	39
Sex: M/W	9/15	5/10
IgE	116.34	14.99
Eosinophils (absolute number) (N.V. < 500 cells per microliter of blood)	350	200
Eosinophils (percent) (N.V. < 7% of the circulating leukocytes)	3.4%	1.8%
VEMS (ml/s)	128	27

**Figure 1:**
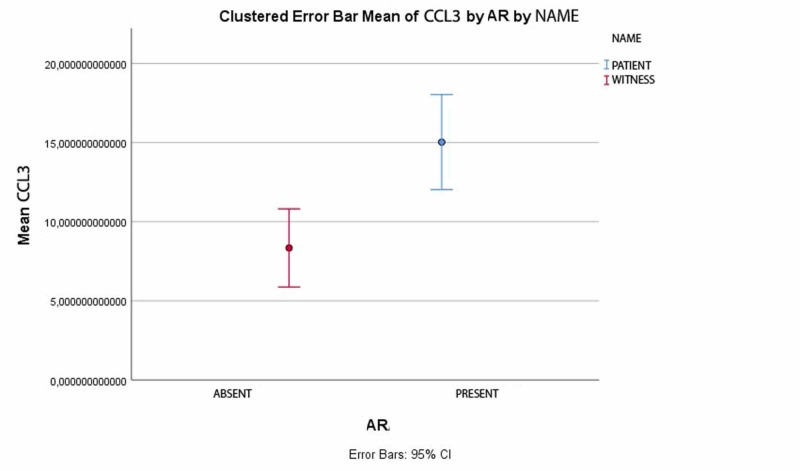
CCL3 values patients versus witness

All participants lived in the city of Bucharest. Total serum IgE levels (a general marker for atopy), the absolute number and percent eosinophils (markers for hypereosinophilia), and maximum expiratory ventilation per second (an indicator of lung airway inflammation) were evaluated. CCL3 concentrations were significantly higher in AR patients than in healthy controls (15.03 ± 7.11 pg/ml versus 8.34 ± 4.46 pg/ml; p = 0.001 by t-test, p = 0.026 by Mann-Whitney U-test) (Tables 2, 3 and Figure 1).

**Table 2:** CCL3 concentrations in AR patients and healthy controls AR: Allergic rhinitis; CCL3: Chemokine ligand 3.

	Group	N	Mean (pg/ml)	Standard deviation	Standard error (mean)
CCL3	AR patients	24	15.031	7.116	1.452
	Healthy controls	15	8.340	4.465	1.152

**Table 3:** Statistical comparisons of CCL3 concentrations in AR patients and healthy controls, as determined by independent samples t-tests AR: Allergic rhinitis; CCL3: Chemokine ligand 3; t: Student t-test; df: Degrees of freedom.

				t-test for equality of means						
				t	df	p-value	Mean difference	Standard error difference	95% Confidence Interval of the difference	
									Lower	Upper
CCL3										
	Equal variances not assumed			3.60	36.990	0.001	6.69	1.85	2.93	10.44

CCL3 concentrations were significantly associated with NMH (p = 0.003 by Mann-Whitney U-test) and correlated with polysensitization (two or more positive skin prick tests per patient; r = 0.325, p = 0.046) and seasonal allergic rhinitis (r = 0.482, p = 0.002; Table 4). The observed correlation between NMH and seasonal allergic rhinitis might be explained by the fact that although SAR has a temporal clinical picture, a subclinical inflammation is presented at cellular levels all years and becomes active when allergens take direct contact with local mast cells.

**Table 4:** Correlations of parameters in this study AR: Allergic rhinitis; NMH: Nasal mucosal hypertrophy; TNSS: Total nasal symptom score; CCL3: Chemokine ligand 3.

		Name	CCL3	Age	AR	Duration of disease	NMH	Seasonality	Sensitization	TNSS
Name	Pearson Correlation	1	-.472^**^	.177	-1.000^**^	-.532^**^	-.556^**^	-.936^**^	-.948^**^	-.741^**^
	Sig. (2-tailed)		.002	.072	.000	.000	.000	.000	.000	.000
	CCL3	Pearson Correlation	-.472^**^	1	.340^*^	.472^**^	.346^*^	.570^**^	.482^**^	.330^*^	.495^**^
		Sig. (2-tailed)	.002		.034	.002	.031	.000	.002	.040	.001
		Age	Pearson Correlation	.177	.340^*^	1	-.177	-.102	-.027	-.154	-.216^*^	-.298^**^
Sig. (2-tailed)			.072	.034		.072	.301	.782	.117	.027	.002
AR			Pearson Correlation	-1.000^**^	.472^**^	-.177	1	.532^**^	.556^**^	.936^**^	.948^**^	.741^**^
	Sig. (2-tailed)		.000	.002	.072		.000	.000	.000	.000	.000
	Duration of disease		Pearson Correlation	-.532^**^	.346^*^	-.102	.532^**^	1	.427^**^	.473^**^	.509^**^	.532^**^
		Sig. (2-tailed)	.000	.031	.301	.000		.000	.000	.000	.000
		NMH	Pearson Correlation	-.556^**^	.570^**^	-.027	.556^**^	.427^**^	1	.601^**^	.528^**^	.564^**^
Sig. (2-tailed)			.000	.000	.782	.000	.000		.000	.000	.000
Seasonality			Pearson Correlation	-.936^**^	.482^**^	-.154	.936^**^	.473^**^	.601^**^	1	.906^**^	.707^**^
	Sig. (2-tailed)		.000	.002	.117	.000	.000	.000		.000	.000
	Sensitization		Pearson Correlation	-.948^**^	.330^*^	-.216^*^	.948^**^	.509^**^	.528^**^	.906^**^	1	.775^**^
		Sig. (2-tailed)	.000	.040	.027	.000	.000	.000	.000		.000
		TNSS	Pearson Correlation	-.741^**^	.495^**^	-.298^**^	.741^**^	.532^**^	.564^**^	.707^**^	.775^**^	1
Sig. (2-tailed)			.000	.001	.002	.000	.000	.000	.000	.000	

CCL3 was also associated with sensitization to grass pollen (p = 0.040) and with patient age (r = 0.495, p = 0.012).

## Discussion

Our finding that serum CCL3 concentrations are higher in patients with AR than in healthy controls is in good agreement with CCL3 concentrations in nasal secretions [3]. The latter results are likely closer to real situations, in that chemokine concentrations were measured directly at the site of allergic reaction rather than in blood samples. Serum CCL3 concentrations may have been affected by systemic inflammatory conditions. To minimize this possibility, we measured important markers of systemic inflammation, including total blood count with differential; concentrations of rheumatoid factor, fibrinogen, and C-reactive protein; and erythrocyte sedimentation rate, and excluded all patients and controls with abnormal results. Measurement of CCL3 and other chemokine levels in nasal secretions is, by far, the most sensitive and specific method to evaluate their presence in allergic rhinitis. But this demands a specialized stuff (nurse, physicians (otorhinolaryngologist), laboratory personal) that is limited to university/research facilities. Measurement of chemokines in serum samples may be performed in many ordinary laboratories around the globe. This technique is affected by the fact that levels of chemokines might be influenced by other systemic conditions.

The association between CCL3 concentration and NMH confirms that CCL3 plays a role in the pathogenesis of chronic inflammation, including respiratory allergies [2, 3]. Moreover, our finding of an association between CCL3 and grass pollen allergy is in agreement with previous results [6, 9]. CCL3 concentration appears to be associated with pollen sensitization, but this observation requires confirmation in future studies [3, 6, 7]. To our knowledge, however, no previous study had reported a correlation between CCL3 concentration and polysensitization to aeroallergens, providing further evidence of the role of CCL3 in the pathogenesis of AR. Several studies have suggested a role for CCL3 in allergen sensitization [3, 6, 7].

The CCL3 gene may be a selective end-point marker in CD34+-progenitor derived dendritic cells exposed to contact allergens and irritants, enabling these cells to differentiate between chemical sensitizers and irritants [14]. We also observed a correlation between CCL3 concentration and TNSS (r = 0.495, p = 0.001). The mean TNSS score in the group of 24 patients was 7.0 ± 3.83, indicating moderate disease. This may indirectly suggest that CCL3 plays a role in the pathogenesis of AR.

Our study has two major limitations. First, the study population was relatively small, which might have affected the statistical power of the study. Secondly, while we tried to eliminate all systemic inflammatory conditions that might influence the levels of CCL3 by using the most important inflammatory markers and keeping only participants with healthy values, we could not exclude a possible infection or inflammation in one or more patients, which constitutes the second major limitation of our study.

## Conclusions

Our study intended to evaluate the role of CCL3 in the pathogenesis of AR. CCL3 presented significant differences between patients with AR and healthy controls. We have shown that the measurement of the levels of CCL3 in blood samples might represent an alternative approach of evaluating chemokines in allergic rhinitis in a non-academic outpatient facility when nasal collection of probes is not feasible. CCL3 also correlated with some aspects of respiratory allergies, such as polysensitization, seasonal allergies, and nasal mucosal hypertrophy. These findings indicate that CCL3 may play a role in AR. Our study implicated a small number of patients and evaluated CCL3 only from blood samples, and therefore, our results should be interpreted with caution. Future studies including a larger number of participants and samples from both the nose and blood may be necessary to confirm the association between CCL3 and AR.
